# Association of *PIP4K2A* Polymorphisms with Alcohol Use Disorder

**DOI:** 10.3390/genes12101642

**Published:** 2021-10-19

**Authors:** Olga Yu. Fedorenko, Ekaterina V. Mikhalitskaya, Valentina A. Toshchakova, Anton J. M. Loonen, Nikolay A. Bokhan, Svetlana A. Ivanova

**Affiliations:** 1 Mental Health Research Institute, Tomsk National Research Medical Center of the Russian Academy of Sciences, 634014 Tomsk, Russia; uzen63@mail.ru (E.V.M.); valia_as@mail.ru (V.A.T.); bna909@gmail.com (N.A.B.); ivanovaniipz@gmail.com (S.A.I.); 2 Division for Control and Diagnostics, School of Non-Destructive Testing and Security, National Research Tomsk Polytechnic University, 634050 Tomsk, Russia; 3 PharmacoTherapy, Epidemiology and Economics, Groningen Research Institute of Pharmacy, University of Groningen, 9713AV Groningen, The Netherlands; a.j.m.loonen@rug.nl; 4 Department of Psychotherapy and Psychological Counseling, National Research Tomsk State University, 634050 Tomsk, Russia; 5 Department of Psychiatry, Addictology and Psychotherapy, Siberian State Medical University, 634050 Tomsk, Russia

**Keywords:** alcohol use disorder, *PIP4K2A*, polymorphisms

## Abstract

Background: Alcohol use disorder (AUD) not only influences individuals and families but also has a lasting social impact on communities at the national level. Dopaminergic neurotransmission is involved in excessive alcohol consumption. Phosphatidylinositol-5-phosphate-4-kinase type 2 α (PIP4K2A) plays an important role in the regulation of ascending dopamine pathways. In this study; we determined possible associations between nine polymorphisms in *PIP4K2A* and AUD in Russian men. Methods: 279 Russian men with AUD were investigated. The control group consisted of 222 healthy men from the general Russian population. Genotyping of DNA samples for nine polymorphic variants of *PIP4K2A* was carried out by the Applied Biosystems™ QuantStudio™ 5 Real-Time PCR System with use of the TaqMan1 Validated SNP Genotyping Assay (Applied Biosystems; CIIIA). Results: Carriage of the *PIP4K2A* rs2230469*TT/T genotype/allele was a relative risk factor for developing AUD in men (*p* = 0.026 and *p* = 0.0084 accordingly). Moreover; men with AUD had a higher frequency of *PIP4K2A* rs746203*T allele (*p* = 0.023) compared to healthy men. Conclusions: For the first time; we demonstrated different *PIP4K2A* polymorphisms to be associated with AUD presumably due to dopamine system modulation resulting from regulation of the lateral habenula.

## 1. Introduction

Alcohol use disorder (AUD) is a serious medical and social burden in most countries of the world. It not only affects individuals and their families but furthermore has a long-lasting impact on social functioning at the national level. According to the World Health Organization (WHO), 3.3 million people worldwide are dying because of excessive alcohol consumption and its consequences every year [1]. The heritability of AUD has been estimated to be approximately 50–60% [2,3]. However, this heritability is largely attributable to alcohol-metabolizing enzymes, as genome-wide associations studies (GWAS) show [4]. This is probably related to the complex nature of the relationship between genotypes and behavioral phenomena such as alcohol abuse. Moreover, numerous candidate-gene and GWAS studies have been considering the possible relationship between variants of genes involved in dopaminergic neurotransmission and alcohol-related mental disorders [3,4,5,6,7,8,9].

Nevertheless, the connection between dopamine-regulated processes and addiction is unmistakably present [10]. The reason that dopamine’s influence on addiction is not straightforward is probably due to the complexity of the neurobiological addiction cycle [10]. According to these authors, alcohol and drug use disorders should be seen as the elaboration of a three-phase cyclic complex determining motivation: binge/intoxication, withdrawal/negative affect, and preoccupation/anticipation. This can also very well be framed within the functioning of evolutionary old parts of the forebrain that are controlled by the habenula [11,12,13]. Dopaminergic neurotransmission plays a role in many places in the structures involved, sometimes with the opposite effect. This alone makes the consequences of genetic changes within dopaminergic neurotransmission less clear.

A potential gene candidate involved in dopaminergic neurotransmission might be the *PIP4K2A* (phosphatidylinositol-5-phosphate-4-kinase type 2 α), which has been associated with the risk of schizophrenia, as well as tardive dyskinesia [14,15,16,17]. To avoid potential confusion, it should be noted that according to the HUGO Gene Nomenclature Committee (HGNC) the previous name of this gene was phosphatidylinositol-4-phosphate 5-kinase, type II, α, and the alias symbol was PIP5KIIA. The currently approved symbol for it is *PIP4K2A*. PIP4K2A plays an important role in the regulation of neuronal excitability and synaptic dopamine neurotransmission via modulation of neuronal KCNQ2/KCNQ3 and KCNQ3/KCNQ5 channels, the EAAT3 glutamate transporter, and GluA1 function [18,19,20]. Therefore, we determined possible associations between nine polymorphisms in *PIP4K2A* and AUD in Russian men.

## 2. Materials and Methods

### 2.1. Patients

Participants were recruited from the addiction department of the Mental Health Research Institute, Tomsk National Research Medical Center (Tomsk NRMC, Tomsk, Russia) (279 Russian men with alcohol use disorder, aged 41 (range: 34–50) years). Inclusion criteria were: a diagnosis of AUD (F10.2) according to ICD-10 [21] and 18–60 years of age. We excluded patients with other comorbid mental disorders and acute somatic diseases. The screening for relevant pathology for in/exclusion of subjects was performed through a clinical assessment on the first day of admission to the addiction department of the Mental Health Research Institute. The control group consisted of 222 healthy male volunteers (aged 26 (range 22–35) years) from the general Russian population.

### 2.2. Genetic Analysis

Blood samples were obtained by antecubital venipuncture after 8 h overnight fasting in EDTA-containing tubes and stored in several aliquots at −20 °C until DNA isolation using the standard phenol-chloroform method. Genotyping of DNA samples of examined persons was carried out for nine single nucleotide polymorphisms (SNPs) of *PIP4K2A* (rs8341, rs746203, rs943190, rs946961, rs1132816, rs1417374, rs10430590, rs2230469 (According to the NCBI SNP database (https://www.ncbi.nlm.nih.gov/snp/, accessed on 2 October 2021): rs10828317 has merged into rs2230469), rs11013052) at The Core Facility “Medical Genomics”, Tomsk NRMC by Applied Biosystems™ QuantStudio™ 5 Real-Time PCR System (Applied Biosystems, Waltham, MA, USA) with the use of the TaqMan1 Validated SNP Genotyping Assay (Applied Biosystems, CIIIA).

### 2.3. Statistical Analysis

Statistical analysis was carried out using SPSS software, release 23.0. The Hardy–Weinberg equilibrium (HWE) of genotypic frequencies was tested by the chi-square test. Pearson’s chi-squared test with Yates’ correction was used for between-group comparisons of genotypic and allelic frequencies at a significance level of *p* < 0.05. Assessment of the association of genotypes and alleles of the studied polymorphic variants with a pathological phenotype (AUD) was carried out using the odds ratio (OR) with a 95% confidence interval for the odds ratio (95% CI).

## 3. Results

The distribution of all genotypes was in accordance with the Hardy–Weinberg equilibrium except for rs946961 and rs10430590 in controls (*p* = 0.003, *p* = 0.0004 respectively). These SNPs were excluded from the analysis. Comparing two groups of men (patients with AUD and healthy control persons), we found significant differences for two out of nine investigated *PIP4K2A* SNPs.

### 3.1. PIP4K2A rs746203

The comparison of studied groups revealed statistically significant differences in the frequencies of the *PIP4K2A* rs746203 alleles distribution between the group of patients with AUD and the control group (*x*^2^ = 5.20, *p* = 0.023) (Table 1). The *PIP4K2A* rs746203*T allele was significantly more frequent in the group of patients with AUD in comparison with the control group (OR = 1.36, 95% Cl = 1.04–1.77).

### 3.2. PIP4K2A rs2230469 (rs10828317)

Carriage of the *PIP4K2A* rs2230469*TT genotype (χ2 = 7.27, *p* = 0.026; OR = 1.42, 95% Cl = 1.05–2.05) and *PIP4K2A* rs2230469*T allele (χ2 = 6.95, *p* = 0.0084; OR = 1.45, 95% Cl = 1.10–1.92) was a relative risk factor for developing AUD in men (Table 2).

We did not find any significant differences for *PIP4K2A* rs8341, rs943190, rs1132816, rs1417374, and rs11013052 (Supplementary Tables S1–S5).

## 4. Discussion

To our knowledge this is the first study of the relationship between *PIP4K2A,* formerly also known as *PIP5K2A,* polymorphism, and AUD. The results of our study indicate that the *PIP4K2A* may have a role in developing AUD. To avoid possible confusion, it should be mentioned that according to the NCBI SNP database (https://www.ncbi.nlm.nih.gov/snp/, accessed on 2 October 2021) the previous nomenclature rs10828317 has merged into the new nomenclature rs2230469. In our study, carriers of the *PIP4K2A* rs2230469*TT/T, and rs746203*T genotypes/alleles were more likely to have AUD. Interestingly, *PIP4K2A* rs2230469*CC genotype was previously found to be a relative risk factor for schizophrenia [16].

Activation of ascending mesolimbic dopaminergic pathways to the ventral striatum may have an important role in causing AUD [22]. The activity of these pathways is under indirect inhibitory control by the lateral habenula (LHb), [9] which is in turn regulated by glutamatergic terminals originating within the pallidal basal ganglia and evaluating the result of reward-seeking and distress-avoiding activities [23,24,25]. Acute and chronic alcohol exposure in animals may modulate the functioning of LHb neurons by altering M-type potassium channels and glutamatergic transmission [26]. PIP4K2A has been disclosed to be a novel signaling element in the regulation of the neuronal KCNQ2/KCNQ3 and KCNQ3/KCNQ5 channels, EAAT3 glutamate transporter, and GluA1 function [18,19,20]. This suggests that functional *PIP4K2A* polymorphisms may affect dopaminergic neurotransmission in response to alcohol exposure and this might contribute to the genetic component of AUD [22].

**Figure 1:**
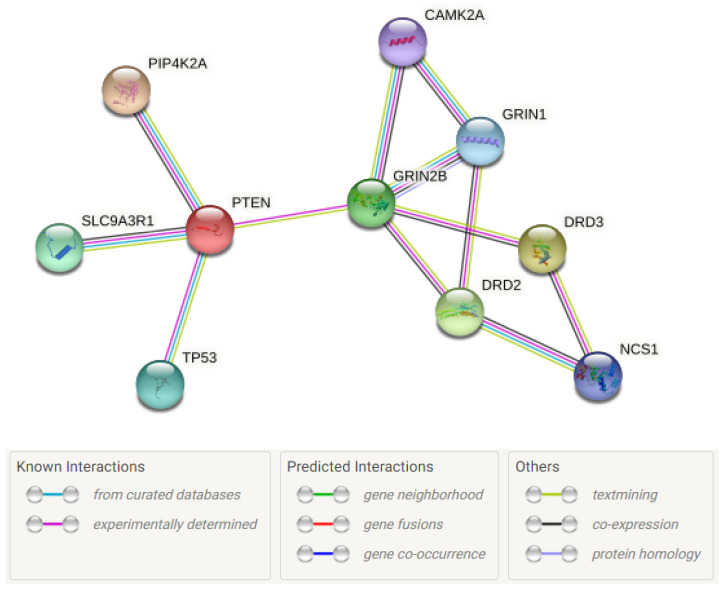
Scheme of possible protein interactions between PIP4K2A and dopamine receptors DRD2, DRD3 created using the https://string-db.org, accessed on 27 September 2021.

Using the https://string-db.org, accessed on 27 September 2021, we have created a scheme of possible protein interactions that depicts possible functional interactions between PIP4K2A and dopamine receptors DRD2, DRD3 via PTEN, and GRIN2B receptor (Figure 1).

Apart from the ventral and dorsal striatum ascending mesencephalic dopaminergic terminals run to the prefrontal cerebral cortex and the temporal lobe (amygdaloid and hippocampal complexes) [27]. A subset of glutamatergic corticostriatal fibers project from the medial prefrontal cortex to the striatal striosomal compartment and from this structure GABAergic medium spiny neurons directly and indirectly (via the LHb) regulate the activity of mesencephalic dopaminergic neurons [28]. It has been demonstrated that these corticostriatal fibers are selectively and causally involved in cost–benefit decision making under approach–avoidance conflict conditions [29]. Individuals suffering from AUD demonstrate difficulty with decision making and impulsivity that may be associated with impaired frontal cortical function [30,31]. Enhancing dopaminergic neurotransmission with a catechol-O-methyl-transferase (COMT) inhibitor was found to reduce alcohol consumption and decrease impulsivity in individuals with AUD [32]. Within the prefrontal cortex, inhibition of COMT has a high impact due to the relative scarcity of the dopamine transporter as another mechanism to eliminate dopamine from the synaptic cleft [33].

The results of our study suggest that *PIP4K2A* polymorphism indirectly supports the involvement of dopaminergic neurotransmission into AUD. The data obtained may provide background for developing the new AUD treatment, namely modulators of the lateral habenula functioning which influences the activity of ascending dopaminergic pathways from the upper brainstem.

### Limitations and Strengths of Our Study

The sample sizes of patients with AUD (*n* = 279) and controls (*n* = 222) are rather limited for a genetic study. Therefore, our findings should be considered preliminary. In addition, the information about personality traits, life history including traumatic experiences of patients, is missing. This is the first study relating polymorphisms of *PIP4K2A* (*PIP5K2A*) to AUD. Our findings advocate replication of our study in an independent population of persons with AUD.

## 5. Conclusions

Our results support a possible role of *PIP4K2A* polymorphism in the mechanisms of alcohol use disorder (AUD) development.

## Figures and Tables

**Table 1:** The comparison of *PIP4K2A* rs746203 genotypes and alleles distribution in men with AUD and healthy men.

Genotypes/ Alleles	Patients with AUD (*n* = 253)	Controls (*n* = 220)	*x*^2^, *p*	OR	95% CI	
CC	34 (13.44%)	39 (17.72%)	5.31 0.07	0.72	0.44	1.19
CT	106 (41.90%)	105 (47.72%)		0.79	0.55	1.14
TT	113 (44.67%)	76 (34.55%)		1.53	1.05	2.22
C	174 (34.39%)	183 (41.59%)	5.20 0.023 *	0.74	0.57	0.96
T	332 (65.61%)	257 (58.41%)		1.36	1.04	1.77

AUD, alcohol use disorder; * statistical significance *p* < 0.05.

**Table 2:** The comparison of *PIP4K2A* rs2230469 genotypes and alleles distribution in men with AUD and healthy men.

Genotypes/ Alleles	Patients with AUD (*n* = 250)	Controls (*n* = 216)	*x*^2^, *p*	OR	95% CI	
CC	20 (8.0%)	33 (15.28%)	7.27 0.026 *	0.48	0.27	0.87
CT	97 (38.8%)	87 (40.28%)		0.94	0.65	1.36
TT	133 (53.2%)	96 (44.44%)		1.42	1.05	2.05
C	137 (27.4%)	153 (35.42%)	6.95 0.0084 *	0.69	0.52	0.91
T	363 (72.6%)	279 (64.58%)		1.45	1.10	1.92

AUD, alcohol use disorder; * statistical significance *p* < 0.05.

## Data Availability

The datasets generated for this study will not be made publicly available, but they are available on reasonable request to Svetlana A. Ivanova (ivanovaniipz@gmail.com), following approval of the Board of Directors of the MHRI, in line with local guidelines and regulations.

## Supplementary Materials

The following are available online at https://www.mdpi.com/article/10.3390/genes12101642/s1, Supplement Table S1. The comparison of PIP4K2A rs8341 genotypes and alleles distribution in men with AUD and healthy men, Supplement Table S2. The comparison of PIP4K2A rs943190 genotypes and alleles distribution in men with AUD and healthy men, Supplement Table S3. The comparison of PIP4K2A rs1132816 genotypes and alleles distribution in men with AUD and healthy men, Supplement Table S4. The comparison of PIP4K2A rs1417374 genotypes and alleles distribution in men with AUD and healthy men, Supplement Table S5. The comparison of PIP4K2A rs11013052 genotypes and alleles distribution in men with AUD and healthy men.
