# Supplementary material for: Association of PIP4K2A Polymorphisms with Alcohol Use Disorder
Source: Genes (Basel). 2021 Oct 19;12(10):1642. doi: 10.3390/genes12101642 (PMC8535504; doi:10.3390/genes12101642)
Supplement: Supplementary file 1 [file genes-12-01642-s001.zip › genes-1397535-supplementary.pdf]

We did not find any significant differences for *PIP4K2A* rs8341, rs943190, rs1132816, rs1417374, and rs11013052 (Supplement tables 1-5).

Supplement Table S1. The comparison of *PIP4K2A* rs8341 genotypes and alleles distribution in men with AUD and healthy men.

**Genotypes/ Alleles**	**Patients with AUD (n=279)**	**Controls (n=223)**	$\chi2$, p	OR	95% CI	
CC	127 (45.52 %)	94 (42.15 %)	$\chi2_1$= 1.46, p_1 = 0.48	1.15	0.80	1.64
CT	117 (41.94 %)	93 (41.70 %)		1.01	0.71	1.44
TT	35 (12.54 %)	36 (16.14 %)		0.75	0.45	1.23
C	371 (66.49 %)	281 (63.00 %)	$\chi2_1$= 1.32, p_1 = 0.25	1.16	0.90	1.51
T	187 (33.51 %)	165 (37.00 %)		0.86	0.66	1.11

Supplement Table S2. The comparison of *PIP4K2A* rs943190 genotypes and alleles distribution in men with AUD and healthy men.

**Genotypes/ Alleles**	**Patients with AUD (n=256)**	**Controls (n=220)**	$\chi2$, p	OR	95% CI	
CC	30 (11.72 %)	33 (15.00 %)	$\chi2_1$= 1.22, p_1 = 0.54	0.75	0.44	1.28
CT	116 (45.31 %)	99 (45.00 %)		1.01	0.71	1.45
TT	110 (42.97 %)	88 (40.00 %)		1.13	0.78	1.63
C	176 (34.38 %)	165 (37.5 %)	$\chi2_1$= 1.00, p_1 = 0.32	0.87	0.67	1.14
T	336 (65.63%)	275 (62.5 %)		1.15	0.88	1.49

Supplement Table S3. The comparison of *PIP4K2A* rs1132816 genotypes and alleles distribution in men with AUD and healthy men.

**Genotypes/ Alleles**	**Patients with AUD (n=254)**	**Controls (n=219)**	$\chi2$, p	OR	95% CI	
AA	148 (58.27 %)	118 (53.88 %)	$\chi2_1$= 2.04, p_1 = 0.36	1.20	0.83	1.72
AG	89 (35.04 %)	79 (36.07 %)		0.96	0.66	1.39
GG	17 (6.69 %)	22 (10.04 %)		0.64	0.33	1.24
A	287 (77.99 %)	315 (71.92 %)	$\chi2_1$= 1.83, p_1 = 0.18	1.22	0.91	1.63
G	81 (22.01 %)	123 (28.08 %)		0.82	0.61	1.09

Supplement Table S4. The comparison of *PIP4K2A* rs1417374 genotypes and alleles distribution in men with AUD and healthy men.

Genotypes/ Alleles	Patients with AUD (n=239)	Controls (n=216)	χ2, p	OR	95% CI	
AA	14 (5.86 %)	22 (10.18 %)	$\chi2_1$= 3.16 p_1 = 0.21	0.55	0.27	1.10
AG	111 (46.44 %)	91 (42.13 %)		1.19	0.82	1.73
GG	114 (47.70 %)	103 (47.68 %)		1.00	0.69	1.45
A	139 (29.08 %)	135 (31.25 %)	$\chi2_1$= 5.08, p_1 = 0.48	0.90	0.68	1.20
G	339 (70.92 %)	297 (68.75 %)		1.11	0.83	1.47

Supplement Table S5. The comparison of *PIP4K2A* rs11013052 genotypes and alleles distribution in men with AUD and healthy men.

Genotypes/ Alleles	Patients with AUD (n=77)	Controls (n=127)	χ2, p	OR	95% CI	
AA	6 (7.79 %)	10 (7.87 %)	$\chi2_1$= 1.174 p_1 = 0.882	0.99	0.34	2.84
AC	25 (32.47 %)	50 (39.37 %)		0.74	0.41	1.34
CC	46 (59.74 %)	67 (52.76 %)		1.33	0.75	2.36
A	37 (24.03 %)	70 (27.56 %)	$\chi2_1$= 0.697, p_1 = 0.706	0.83	0.52	1.32
C	117 (75.97 %)	184 (72.44 %)		1.20	0.76	1.91
